# Low incidence of acute actionable imaging findings in emergency department patients imaged for vertigo: Retrospective analysis and proposed guidelines

**DOI:** 10.1007/s10140-025-02426-2

**Published:** 2025-12-22

**Authors:** Raven Spencer, Jason Gandhi, Justin Tepe, Charles Li, Matthew Kulzer, John O’Neill, Laura Eisenmenger, Michael Goldberg, Aichi Chien, Warren Chang

**Affiliations:** 1 Allegheny Health Network, 320 E. North Ave, Pittsburgh, PA 15201 USA; 2 UCLA Health, 757 Westwood Plaza, Los Angeles, CA 90095 USA; 3 UW Health, 600 Highland Avenue, Madison, WI 53792 USA

**Keywords:** Stroke, Vertigo, CT, MRI, Emergency Department

## Abstract

**Purpose:**

To quantify the diagnostic yield of neuroimaging in adult emergency department (ED) patients presenting with vertigo, and to identify clinical predictors of acute central pathology that can inform imaging decisions.

**Methods:**

This retrospective study reviewed all neuroimaging examinations performed for vertigo at 14 EDs within our health network between May 2016 and January 2025. Adult ED patients (*n*=4,135; mean age 62.5 years; 62% female) who underwent imaging (*n*=5,445 exams, approximately 89% CT and 11% MR) were included. Imaging exams with potentially clinically relevant findings were flagged for further review (*n*=291 exams and patients); these patients were separated into four separate groups based on their imaging findings: 1) acute actionable contributory to vertigo, 2) acute actionable non-contributory to vertigo, 3) non-acute actionable, or 4) non-actionable. Vertigo quality (constant, intermittent/resolved spontaneously, no vertigo), acuity, neurological examination (including cerebellar signs and the Head-Impulse, Nystagmus, and Test-of-Skew [HINTS] exam), and intervention rates were analyzed within these subgroups using Fisher’s exact and chi-square tests.

**Results:**

Of 5,445 exams, 291 (5.3%) were flagged with potentially relevant imaging findings. Of these exams, only 115 (2.1%) yielded actionable findings, and just 65 (1.2%) revealed acute central causes contributing to vertigo. In patients with positive imaging findings, constant vertigo was strongly associated with acute contributory pathology (98.5% in this group vs. 6.0% in other groups, *p*<0.0001). Acute onset was more frequent in acute contributory cases (63.1% vs. 40.8%, *p*=0.0006), as were abnormal HINTS or cerebellar signs (44.6% vs. 6.0%, *p*<0.0001). Most patients with acute contributory findings received specialty consultations resulting in intervention (95.4%). Intermittent or resolved vertigo was commonly seen in patients with benign peripheral diagnoses.

**Conclusion:**

Neuroimaging frequently yields normal results in ED vertigo cases; acute actionable central findings deemed contributory to vertigo are rare. Only approximately 2% of patients had acute actionable imaging findings and only 1.3% had a stroke. In patients with acute actionable imaging findings, clinical features—especially constant vertigo, acute onset, and abnormal neurological exam—are strongly associated with central causes and should guide selective imaging in the ED.

**Graphical Abstract:**

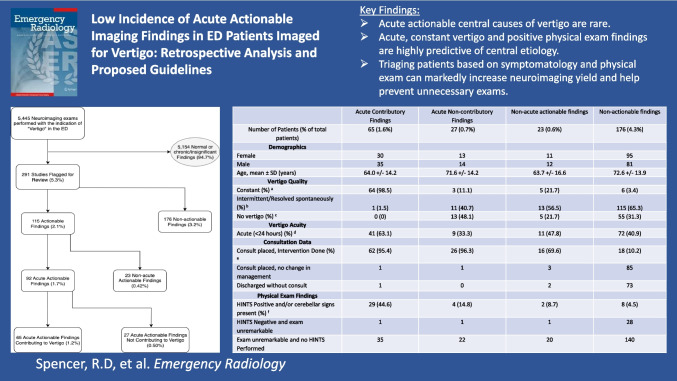

## Introduction

Vertigo is a common complaint in emergency departments (ED), accounting for 2–4% of adult ED visits in the United States [1, 2]. Accurate and timely differentiation between benign peripheral vestibular disorders and serious central nervous system pathologies—such as ischemic stroke, hemorrhage, or neoplasm—is imperative to prevent significant morbidity [3, 4]. However, the broad differential diagnosis, often nonspecific symptoms, and overlap between vertigo and the more ambiguously defined dizziness can complicate evaluation in the acute setting [5].

Neuroimaging, including CT, CTA, and MRI, is frequently performed to exclude central causes of vertigo, but the diagnostic yield is consistently low in ED patients. Large observational studies and meta-analyses estimate acute actionable pathology in only 3–5% for CT and 12–21% for MRI, with the overall prevalence between 1.5% and 8% [3, 4, 6–10]. Despite limited yield, imaging is performed in up to 35% of vertigo-related ED presentations [11, 12]. The combination of high imaging utilization and low diagnostic yield results in substantial healthcare costs and exposes patients to risks of radiation, incidental findings, and unnecessary interventions [13, 14].

The current challenges have encouraged efforts to improve clinical risk stratification and imaging stewardship. The Head-Impulse, Nystagmus, and Test-of-Skew (HINTS) examination has demonstrated sensitivity exceeding 95% for distinguishing central from peripheral causes in acute vestibular syndrome, potentially outperforming early MRI in experienced hands [15–17]. More recent clinical algorithms and risk scores, including the TriAGe+ and Sudbury Vertigo Risk Scores, integrate patient demographics, clinical data, and bedside findings to guide imaging decisions and have shown promise in external validation [18, 19].

Contemporary practice guidelines—including the 2023 American College of Radiology Appropriateness Criteria and the GRACE-3 Guidelines—emphasize selective neuroimaging in vertigo patients, advocating for imaging primarily in cases with focal neurological deficits, vascular risk factors, or inability to confidently exclude central etiologies through bedside evaluation [20, 21]. Acute vestibular syndrome, defined as acute and constant vertigo, is closely associated with central pathology in the ER literature [20, 21]. A diagnostic strategy focused on the timing, triggers, and associated symptoms of vertigo now underpins guideline-based assessment, supported by data that detailed history and neurological examination remain cornerstones of neuroimaging triage [20, 21].

Real-world data regarding imaging yield, risk stratification tools, and guideline compliance remain limited. Our study systematically examined nearly a decade of neuroimaging studies for ED patients presenting with vertigo across a large health system, quantifying diagnostic yields for both acute and non-acute findings, and evaluating the clinical features and outcomes associated with actionable diagnoses. To our knowledge, this study represents the most extensive system-level retrospective analysis of neuroimaging for vertigo. Our data underscore the persistent paradox of low imaging yield despite high utilization and emphasize the need to align imaging practice with evidence-based risk stratification and contemporary guidelines.

## Materials and methods

This study was performed under the supervision of the local Institutional Review Board. Informed consent was waived due to the retrospective nature of the study.

Our network includes fourteen hospitals with emergency departments across a large multi-state geographic area. Retrospective analysis was performed between May 2016 and January 2025. The patient cohort was selected by performing an advanced search within our PACS, selecting all adult ED patients who underwent neuroimaging with the indication of vertigo. Studies without an indication of vertigo (dizziness, lightheadedness, etc.) were not included in the study. Patients were included in the study if they were seen in a network ED and were either imaged in the ED or had imaging ordered in the ED by an ED or admitting provider which was performed while in inpatient or observation status. Patients who had imaging ordered after admission or after inpatient consultation were not included in the study.

**Fig. 1:**
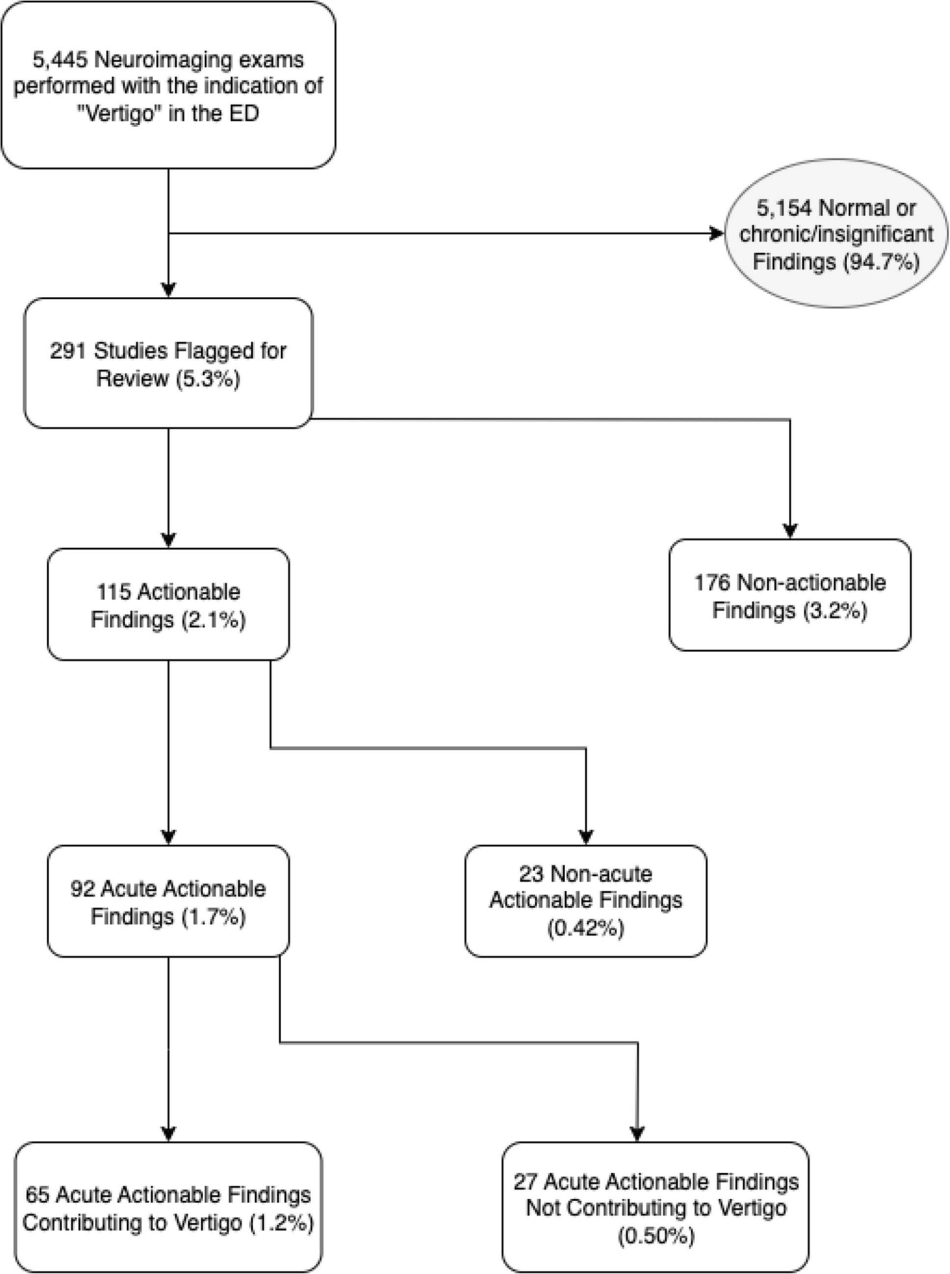
Study flowchart depicting selection and classification of 5,445 neuroimaging exams performed with the indication of vertigo in the emergency department, highlighting the proportion of actionable versus non-actionable findings

A total of 4135 patients (2576 females, 1559 males, average age 62.5 years) underwent neuroimaging for the indication of “vertigo” (Fig. 1 and Table 1). 5445 total exams were performed during 4238 unique patient encounters.

**Table 1:** Distribution of neuroimaging studies ordered with the indication of vertigo (*N* = 5,445) categorized by modality and percentage flagged for further review

	All Exams	Flagged for Review
Total	5,445 (100%)	291 (5.3%)
CT head/face/temporal bone	2,630 (48.3%)	36 (0.7%)
CT Angiogram	2,143 (39.4%)	198 (3.6%)
MRI Brain/IAC	457 (8.4%)	45 (0.8%)
MR Angiogram	140 (2.6%)	7 (0.1%)
MR or CT Venogram	19 (0.3%)	0 (0%)
Other	56 (1.0%)	5 (0.09%)

All neuroimaging reports were reviewed by board certified neuroradiologists and neurologists; all studies with potentially clinically relevant findings were flagged for further review. Studies were flagged as potentially clinically relevant if they demonstrated: (1) new (or at least moderate) vertebrobasilar arterial stenosis, (2) equivocal findings on initial imaging (for example, if there was questionable gray-white differentiation loss on CT), (3) any acute pathology (intracranial hemorrhage, infarct, vessel occlusion, etc.), or (4) any other new abnormal findings.

**Fig. 2:**
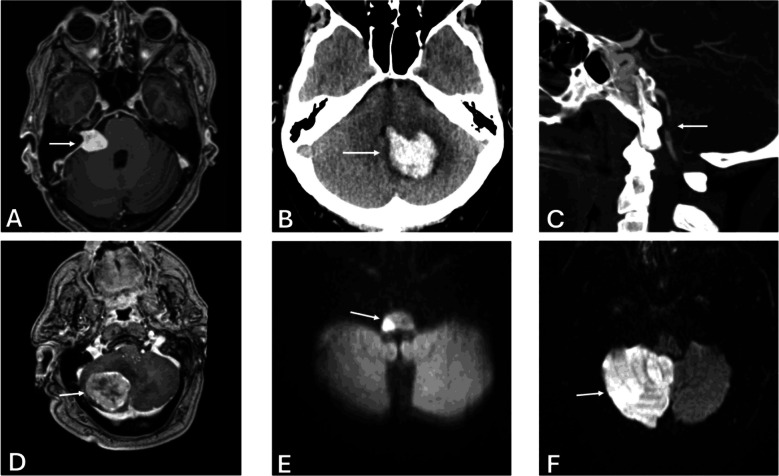
Neuroimaging examples of actionable pathologies detected in patients presenting with vertigo. (**A**) Right-sided vestibular schwannoma (axial contrast-enhanced T1-weighted MRI); (**B**) Left cerebellar intraparenchymal hemorrhage causing fourth ventricular compression (non-contrast CT); (**C**) Occlusive thrombus in left vertebral artery V4 segment (sagittal CTA maximal intensity projection); (**D**) Right cerebellar metastasis with mass effect (axial contrast-enhanced T1-weighted MRI); (**E**) Right lateral medullary infarct (axial DWI MRI); (F) Extensive right cerebellar infarct (axial DWI MRI)

For each flagged study, all images were reviewed by a board certified neuroradiologist. Additional information was gathered from the patient’s electronic medical record (EMR). This information included, but was not limited to, patient demographics, past medical history, family history, vertigo quality (constant, intermittent or positional, or resolved spontaneously) vertigo acuity/duration, imaging modality used (CT, MRI, CTA, MRA, etc.), specialty consultations (neurology, neurosurgery, and otolaryngology), physical examination findings performed by an emergency physician and/or specialist consultant (HINTS examination, focal cerebellar sign assessment, etc.), whether or not in-hospital interventions were performed, and disposition. Interventions, if performed, included medical management (initiation of dual antiplatelet therapy, anticoagulation, thrombolytic therapy, hypertension control, etc.) or procedures (external ventricular drain placement, craniotomy, etc.). Subsequent imaging, if performed, was also reviewed to confirm initial imaging diagnosis when pathology was present. Of note, a subset of patients who were imaged for the indication of vertigo subsequently denied vertiginous symptoms on further clinical assessment – these occurrences were also recorded in detail (Fig. 2).

Once the flagged studies and EMR were reviewed, each flagged patient was separated into one of the four following categories based on their neuroimaging findings: (1) acute actionable findings contributing to vertigo (acute contributory findings), (2) acute actionable findings not contributing to vertigo (acute non-contributory findings), (3) non-acute actionable findings, and (4) non-actionable findings. Acute actionable findings were defined as imaging abnormalities requiring urgent management (i.e., required immediate intervention and/or additional diagnostics prior to discharge), whereas non-acute actionable findings required further workup and/or could be feasibly managed in a future hospital or outpatient encounter. Acute actionable findings were considered contributory to vertigo if they involved the posterior fossa, vertebrobasilar vasculature, or the vestibular cortex. Acute, actionable non-contributory findings were defined as cases in which patients had an acute imaging abnormality that resulted in admission or a change in management that was not thought to be contributing to their symptoms of vertigo. These included acute infarcts, hemorrhage, and neoplasm involving the anterior circulation that did not involve the vestibular cortex, among other findings. Findings were considered non-actionable if the patient was discharged from the ED without consultation or change in management regarding their imaging findings; in many of these cases, peripheral vertigo was diagnosed.

In addition to categorizing patients by imaging findings into four groups as described above, we also performed stratification of the same flagged patients based on their symptoms (specifically, vertigo quality) to explore the relationship between clinical presentation and underlying central pathology. To do this, patients were grouped according to vertigo symptom quality as follows: (1) those reporting constant vertigo (constant), (2) those with intermittent or positional vertigo or vertigo that resolved spontaneously (intermittent/resolved spontaneously), and (3) those who denied vertigo on subsequent assessment (no vertigo). Patients were also classified as having chronic or acute presentation of vertigo, with acute vertigo being defined as symptom onset < 24 hours prior to presentation.

Continuous variables were reported as the mean ± standard deviation. Regarding statistical analyses, comparisons between categorical variables were performed using Chi-square or Fisher’s exact tests; post-hoc pairwise comparisons were corrected for multiple testing using Holm-Bonferroni adjustments. Relative risks with 95% confidence intervals were also calculated and reported when significant.

## Results

Of the 5,445 imaging studies, 291 studies (5.3%) representing 291 unique patients (7.0%) were flagged for further review, and 5154 studies (94.7%) representing 3844 unique patients (93.0%) demonstrated normal or chronic non-significant imaging findings.

Of the flagged studies, 115 (2.1% of total) had actionable findings. These included acute actionable findings in 92 exams (1.7% of total), subdivided into 65 exams (1.2% of total) with acute contributory findings, and 27 exams (0.5% of total) with acute non-contributory findings. Acute contributory findings consisted of brainstem or cerebellar infarcts (51), posterior fossa hemorrhage (5), posterior fossa neoplasm with mass effect (6), and vertebral artery dissection (3). Acute non-contributory findings included non-vertiginous infarcts (supratentorial infarcts not involving the vestibular cortex, 16), non-vertiginous hemorrhage (supratentorial parenchymal, subdural, or small subarachnoid hemorrhage, 7), supratentorial neoplasm with mass effect (1), active demyelination (1), Creutzfeldt-Jakob Disease (1), and hydrocephalus (1) (Table 2).

**Table 2:** Classification of exams flagged for review (*N* = 291), including breakdown of acute and non-acute actionable findings, and non-actionable diagnoses such as peripheral vertigo and unrelated hospital diagnoses

	Number of Exams	% of Total Exams
Total Number of Exams	5,445	100
Exams Flagged for Review	291	5.3%
Total Actionable Findings	115	2.1%
Acute Actionable Findings	92	1.7%
Acute Contributory Findings	65	1.2%
Posterior fossa/vestibular cortex infarct	51	0.9%
Posterior fossa hemorrhage	5	0.09%
Posterior fossa neoplasm with mass effect	6	0.09%
Vertebral Artery Dissection	3	0.06%
Acute Non-contributory Findings	27	0.50%
Non-vertiginous infarct	16	0.29%
Non-vertiginous hemorrhage	7	0.13%
Active Demyelination/CJD/Neoplasm/Hydrocephalus	4	0.07%
Non-acute Actionable Findings	23	0.42%
Vertebrobasilar artery insufficiency	14	0.25%
Posterior fossa neoplasm without mass effect	1	0.02%
Vestibular/facial nerve schwannoma	2	0.04%
Labyrinthitis	2	0.04%
Inactive Demyelination/Susac Syndrome	3	0.06%
Semicircular canal dehiscence	1	0.02%
Non-actionable Findings	176	3.2%
BPPV/Peripheral Vertigo	100	1.8%
Orthostatic hypotension/vasovagal/nonspecific dizziness	34	0.62%
Other (unrelated hospital diagnosis)	42	0.77%

There were 23 patients with non-acute actionable findings (0.4% of total), most of which were vertebrobasilar artery stenosis/insufficiency with subsequent transient ischemic attack (TIA, 14), posterior fossa neoplasms without mass effect (1), vestibular schwannomas (2), labyrinthitis (2), inactive demyelinating disease (2), Susac syndrome (1), and superior semicircular canal dehiscence (1). The non-actionable findings group (176, 3.2% of total) was comprised of patients ultimately diagnosed with peripheral vestibular disorders such as benign paroxysmal positional vertigo (BPPV, 100), orthostatic hypotension or nonspecific dizziness (34), and other unrelated diagnoses (42).

Data analyses revealed that vertigo quality significantly differed among the imaging finding groups (Fisher’s exact test *p* < 0.0001). Patients with acute contributory findings almost universally presented with constant vertiginous symptoms (98.5%) versus only 6.0% of patients across the other three groups (*p* < 0.0001, RR = 16.4, 95% CI: 10.0–26.9.0.9). Conversely, patients with acute contributory findings were very unlikely to have intermittent symptoms or symptoms that resolved spontaneously (1.5% vs. 61%, *p* < 0.0001) or report no vertigo at time of assessment (0% vs. 33%, *p* < 0.0001). By comparison, most patients in the non-actionable group had intermittent symptoms (65.3%) or reported no vertigo (31.3%) (Table 3).

**Table 3:** Clinical comparison between groups of patients (*N* = 291) with abnormal imaging findings based on vertigo relevance and acuity of imaging findings: acute contributory findings, acute non-contributory findings, non-acute actionable findings, and non-actionable findings. Includes demographic, clinical, physical exam, consultation, and admission data

	Acute Contributory Findings	Acute Non-contributory Findings	Non-acute actionable findings	Non-actionable findings
Number of Patients (% of total patients)	65 (1.6%)	27 (0.7%)	23 (0.6%)	176 (4.3%)
Demographics				
Female	30	13	11	95
Male	35	14	12	81
Age, mean ± SD (years)	64.0 +/- 14.2	71.6 +/- 14.2	63.7 +/- 16.6	72.6 +/- 13.9
Vertigo Quality				
Constant (%) a	64 (98.5)	3 (11.1)	5 (21.7)	6 (3.4)
Intermittent/Resolved spontaneously (%) b	1 (1.5)	11 (40.7)	13 (56.5)	115 (65.3)
No vertigo (%) c	0 (0)	13 (48.1)	5 (21.7)	55 (31.3)
Vertigo Acuity				
Acute (<24 hours) (%) d	41 (63.1)	9 (33.3)	11 (47.8)	72 (40.9)
1–7 days	23	2	3	33
>7 days	1	2	4	15
No Vertigo	0	13	5	55
Consultation Data				
Consult placed, Intervention Done (%) e	62 (95.4)	26 (96.3)	16 (69.6)	18 (10.2)
Consult placed, no change in management	1	1	3	85
Discharged without consult	1	0	2	73
Transferred prior to consult/Left AMA	1	0	2	0
Physical Exam Findings				
HINTS Positive and/or cerebellar signs present (%) f	29 (44.6)	4 (14.8)	2 (8.7)	8 (4.5)
HINTS Negative and exam unremarkable	1	1	1	28
Exam unremarkable and no HINTS Performed	35	22	20	140
Admission Data				
Discharged from ED	0	0	5	49
If admitted, Length of Hospital Stay	5.5 +/- 8.7 days	2.8 +/- 2.0 days	2.4 +/- 2.0 days	2.2 +/- 1.9 days

a: Constant vertigo was significantly more common in the acute contributory group (98.5%) compared with other groups combined (6.0%), Fisher's exact *p*<0.0001, RR=16.4 (95% CI 10.0–26.9).

b: Intermittent/resolved vertigo was significantly less common in the acute contributory group (1.5%) compared to the other groups combined (61.0%), Fisher's exact *p*<0.0001, OR=0.009 (95% CI 0.001–0.07).

c: 0% of the acute contributory patients reported no vertiginous symptoms on subsequent clinical assessment compared to 33% in the other groups combined, *p*<0.0001.

d: Acute vertigo (<24 hrs) was significantly more frequent in acute contributory patients (63.1%) vs other groups combined (40.8%), *p*=0.0006, RR=1.58 (95% CI 1.26–1.99); this difference was most notable when compared to the non-actionable group, with pairwise comparison *p*=0.002

e: Consultations resulting in interventions were more common in acute contributory (95.4%) vs overall (34.2%), RR=2.8 (95% CI 2.2–3.6), *p*<0.0001; particularly vs the non-actionable group (10.6%), RR=9.0 (95% CI 5.6–14.3). There was no difference in intervention rates between the two acute actionable groups, which was an expected finding.

f: Abnormal HINTS/cerebellar signs were strongly linked to the acute contributory group (44.6%) vs others combined (6.0%), RR=7.4 (95% CI 4.3–12.7), *p*<0.0001; paired comparison with the acute non-contributory group was also significant (44.6% vs 14.8%), RR=3.0 (95% CI 1.0–8.5), *p*=0.04.

The presence of acute contributory findings was also associated with vertigo acuity of less than 24 hours onset. Specifically, 63.1% (41/65) of patients with acute contributory findings presented with acute vertigo of less than 24 hours compared to 40.8% in the other three groups combined (*p* = 0.0006, RR = 1.58, 95% CI: 1.26–1.99.26.99).

Neurological examination findings further corroborated this stratification. Among those with acute contributory findings, 44.6% had abnormal HINTS examination and/or focal cerebellar signs documented compared to 6.0% across the three other groups (*p* < 0.0001, RR = 7.4, 95% CI: 4.3–12.7). Paired analysis comparing the acute contributory findings group to the acute non-contributory findings group demonstrated a significantly higher prevalence of abnormal examination findings in the contributory group (44.6% vs. 14.8%; *p* = 0.04, RR = 3.0, 95% CI: 1.0–8.5). Of note, HINTS was explicitly documented in only 46.2% of acute contributory patients and 25.4% of all flagged patients (Table 3).

Specialist consultation patterns and intervention rates differed across the imaging findings groups. Nearly all patients with acute contributory findings received neurology, neurosurgery, or otolaryngology consultations resulting in an acute change in management (62/65, 95.4%), a significantly higher proportion compared with all other groups combined (34.2%, *p* < 0.0001, RR = 2.8, 95% CI: 2.2–3.6.2.6). This difference was most profound when compared to the non-actionable group (95.4% vs. 10.6%, *p* < 0.0001, RR = 9.0, 95% CI: 5.6–14.3.6.3). Intervention rates were similarly high in the acute actionable non-contributory group (96.3%).

When the patients flagged with potentially clinically relevant imaging findings were instead stratified by vertigo quality (Table 4), our data showed that patients in this subgroup who presented with constant vertigo were more likely to harbor acute actionable central pathology, including infarcts, intracranial hemorrhage, neoplasm with mass effect, and vertebral artery dissection (89.2%) when compared with other patients within this subgroup with intermittent/resolved vertigo (7.7%; *p* < 0.0001, RR = 11.6, 95% CI: 7.3–18.3.3.3) or those denying vertigo (15.6%; *p* < 0.0001, RR = 5.7, 95% CI: 3.4–9.3.4.3). In contrast, intermittent vertigo or vertigo that resolved spontaneously was most often associated with peripheral etiologies, with two-thirds of this group diagnosed with BPPV (66.4%) compared with only 6.0% of patients in the constant group (*p* < 0.0001, RR = 11.1, 95% CI: 5.0–24.7.0.7).

**Table 4:** Comparisons of patient characteristics and outcomes stratified by vertigo symptom quality: Constant, intermittent/resolved spontaneously, or no vertigo. Analyses include demographics, physical exam, consultation outcomes, and central versus peripheral etiologies

	Constant	Intermittent/Resolved Spontaneously	No Vertigo
Number of Patients (*N* = 291)	78	140	73
Demographics			
Female	36	81	32
Male	42	59	41
Age, mean ± SD (years)	65.1 +/- 14.1	72.1 +/- 13.8	70.7 +/- 15.8
Physical Exam Findings			
Abnormal HINTS and/or Cerebellar Signs ^c^	33 (42.3%)	36 (25.7%)	5 (6.8%)
Unremarkable exam	45	104	68
Consultation Data			
Discharged without consult	3 (3.8%)	40 (28.6%)	34 (46.6%)
Consulted, no change in management	1	69	20
Consulted, intervention done ^d^	74 (94.9%)	31 (22.1%)	19 (26.0%)
Diagnoses			
Acute Diagnoses ^a^			
Acute/subacute Infarct	55 (70.5%)	5 (3.6%)	8 (11.0%)
Intracranial Hemorrhage	5 (6.4%)	1 (0.7%)	2 (2.7%)
Intracranial Neoplasm/Cerebral Edema	8(10.3%)	2 (1.4%)	n(1.4%)
Vertebral artery dissection	2 (2.6%)	1 (0.7%)	0 (0%)
Non-Acute Diagnoses			
Demyelination	0	2	2
TIA/Hypertensive Urgency	3	17	4
BPPV/Peripheral Vertigo ^b^	5 (6.4%)	95 (67.9%)	---
Non-vertigo Diagnosis	0	17	56

a: Patients presenting with constant vertigo were significantly more likely to have acute central pathology (infarct, hemorrhage, neoplasm, or vertebral arterial dissection) compared with those having intermittent/resolved vertigo (89.2% vs. 7.7%, Fisher exact test, *p*<0.0001; risk ratio [RR] 11.6, 95% confidence interval [CI] 7.3–18.3) or no vertigo (89.2% vs. 15.6%, *p*<0.0001; RR 5.7, 95% CI 3.4–9.3).

b: Intermittent/resolved vertigo was strongly associated with peripheral vestibular etiologies, with 66.4% of those patients being diagnosed with BPPV compared to 6.0% in the patients presenting with constant vertigo; Fisher exact test, *p*<0.0001; RR 11.1, 95% CI 5.0–24.7).

c: Abnormal HINTS/cerebellar signs were more common in constant vertigo group (42.3%) compared with intermittent/resolved vertigo group (25.7%, RR 1.65, 95% CI 1.02–2.67, *p*=0.04) and no vertigo group (6.8%, RR 6.23, 95% CI 2.52–15.4, *p*<0.0001

d: Consultations with interventions occurred most frequently in constant vertigo group (94.9% vs. 22.1% intermittent/resolved, RR 4.3, 95% CI 3.0–6.2, *p*<0.0001; vs. 26.0% no vertigo, RR 3.7, 95% CI 2.3–6.1, *p*<0.0001)

## Discussion

Most neuroimaging studies in our cohort revealed normal or clinically non-significant findings (94.7%), with only 1.2% of exams having acute central pathology contributing to vertigo. This low incidence—particularly of acute posterior fossa infarcts—aligns with prior literature describing similarly low rates of acute central pathology in ED patients presenting with vertigo [4, 6–8, 22–24]. These findings reaffirm the limited diagnostic yield of neuroimaging in patients presenting with vertigo, supporting the case for more selective imaging based on risk stratification [9, 10].

Our data highlights the diagnostic value of vertigo symptom quality and acuity. Within the subgroup of patients flagged with potentially clinically relevant imaging findings, those with constant vertigo and symptom onset within 24 hours were more likely to harbor acute contributory imaging pathology, whereas patients with chronic, intermittent, or spontaneously resolved symptoms were more likely to have peripheral etiologies such as BPPV. These clinical distinctions echo prior work on the diagnostic importance of timing and triggers in differentiating acute vestibular syndrome from episodic or positional vertigo [5, 23].

Among existing studies, our cohort size surpasses previous reports evaluating neuroimaging yield in emergency department patients presenting with vertigo. For example, Happonen et al. analyzed 1,169 emergency brain MRIs to assess imaging outcomes in dizziness and vertigo, representing the next largest cohort identified in the literature [8]. Other multicenter or risk score validation studies, such as Yu et al. (TriAGe+ Score; *N*=498) and Ohle et al. (Sudbury Vertigo Risk Score; *N*=2078), have contributed important insights into risk stratification but included fewer patients and often focused exclusively on imaging outcomes or diagnostic accuracy of specific modalities [18, 19]. In contrast, our study not only provides the most extensive system-level analysis to date but also incorporates detailed clinical characterization by systematically examining presenting symptoms, examination findings, and demographic variables in relation to acute neuroimaging diagnoses. This comprehensive approach offers greater granularity and generalizability, enabling more nuanced triage recommendations and optimizing imaging stewardship for vertigo-related ED presentations.

Neurological examination findings further stratified risk in the patients with potentially relevant imaging findings. Abnormal HINTS exam and/or focal cerebellar signs were much more common in patients with acute contributory findings compared to the other groups, including patients with acute non-contributory findings. Conversely, patients with negative or unremarkable examinations were far less likely to harbor actionable causes of vertigo. These results reinforce the established high sensitivity of the HINTS exam for posterior circulation stroke [15–17]. Consultation and intervention patterns paralleled these findings. Nearly all patients with acute contributory imaging pathology underwent specialty consultation and acute changes in management (95.4%), compared with only 34.2% of all others and just 10.2% of those with non-actionable findings. Importantly, rates of acute change in management were comparably high among the acute non-contributory group, which is an expected observation given that acute central imaging findings—even if not explanatory for vertigo—frequently prompt specialty engagement and management.

Re-stratifying patients instead by vertigo quality symptom profile, as demonstrated in Table 4, further underscored the diagnostic importance of clinical presentation. In patients with potentially clinically relevant imaging findings, constant vertigo was highly predictive of acute central pathology, while intermittent or positional vertigo was strongly associated with peripheral etiologies such as BPPV. This bidirectional validation (pathology → symptoms in Table 3; symptoms → pathology in Table 4) strengthens the evidence that symptom quality alone offers meaningful predictive value in triage, a finding that may help clinicians decide which patients warrant urgent advanced neuroimaging.

While age and cardiovascular or stroke risk factors traditionally inform overall stroke risk, these variables fall outside the specific focus of our study, which aims to identify bedside features that stratify patients requiring urgent neuroimaging for vertigo itself. It is important to acknowledge that advanced age and multiple vascular risk factors may nonetheless warrant imaging when clinical concern exists for alternative diagnoses unrelated to vertigo. Our analysis strongly suggests that among patients presenting with true vertiginous symptoms and potentially relevant imaging findings, abrupt and constant vertigo, symptom onset within 24 hours, and the presence of positive HINTS or focal cerebellar signs on neurological examination are strongly associated with acute contributory pathology. Conversely, patients in this cohort that had chronic, intermittent, or positional vertigo, or symptoms that have resolved, are less likely to harbor acute central causes and may not require immediate imaging. This approach aligns with current guidelines emphasizing targeted clinical evaluation and may enhance diagnostic accuracy while optimizing resource utilization in the emergency department.

Beyond the associations identified in our cohort, our findings also illustrate persistent diagnostic challenges in the evaluation of vertigo in the emergency department. First, patient-reported symptomatology is often imprecise. Prior studies have shown that many patients struggle to distinguish true vertigo from nonspecific dizziness, lightheadedness, or orthostatic complaints, leading to frequent mislabeling and potential over-utilization of imaging [5, 25]. In our cohort of patients with potentially relevant imaging findings, more than one fourth of patients denied vertiginous symptoms on subsequent reassessment despite “vertigo” being the original documented imaging indication. This underscores the need for more structured, targeted questioning emphasizing timing and triggers to improve diagnostic accuracy and avoid unnecessary neuroimaging.

Second, although abnormal neurologic findings were more prevalent among patients with acute contributory pathology, fewer than half of these patients had documented neurologic exam abnormalities. Only 44.6% of the acute contributory subgroup had documentation of abnormal HINTS and/or focal cerebellar signs, despite prior evidence that such findings strongly predict posterior circulation stroke [15–17]. This mirrors other series reporting that 40–60% of patients with posterior fossa pathology have abnormal bedside exam findings [17, 26]. Moreover, HINTS was explicitly documented in only 25.4% of cases flagged for review — paralleling prior demonstrations of underuse and inconsistent application of this exam in real-world ED practice [27–31]. These observations highlight the dual challenges of exam sensitivity and provider variability in documentation, reinforcing the need for more consistent implementation of validated bedside tools for acute vestibular syndrome.

The retrospective design of this study introduces several inherent limitations. As our patient selection relies on existing clinical and imaging records, it is susceptible to practice heterogeneity and incomplete or inconsistent documentation. Dependence on recorded clinical examination findings led to underestimation of bedside physical exam utilization, as previously discussed. Additionally, some patients received consultations, admissions, or therapies for reasons unrelated to acute imaging findings, potentially confounding downstream management analyses. For example, patients admitted with alternative diagnoses such as hypertensive urgency or trauma may have received specialty consultations and/or medication adjustments that were not directly attributable to imaging results. Furthermore, patients transferred to other institutions for intervention had incomplete follow-up, leading to underestimation.

Another limitation of our study is that detailed chart review of presenting symptoms and neurological examinations was conducted only for patients flagged with new or concerning imaging findings. The decision to conduct an extensive chart review only on these patients was intended to focus on potentially actionable imaging findings, rather than clinical outcomes for all patients presenting to the ED with vertigo. As a result, our analysis does not include symptom data for the larger cohort of patients with negative imaging, which limits the generalizability of symptom-imaging associations observed. Consequently, while our results suggest that constant, acute vertigo and abnormal neurological exam increase the likelihood of identifying acute central pathology, this relationship cannot be conclusively extended to all ED patients presenting with vertigo in our study. That being said, numerous clinical studies in the ER literature [15, 17–21] have identified acute vestibular syndrome as being highly associated with acute central pathology. Future prospective studies encompassing both positive and negative imaging groups could further confirm high-risk clinical features and more accurately inform neuroimaging triage strategies.

In conclusion, while acute central causes of vertigo remain rare in ED patients undergoing neuroimaging, careful integration of symptom quality, symptom acuity, and bedside neurological examination can significantly improve the identification of high-risk patients. Specifically, those presenting with constant vertigo of < 24 h duration and abnormal HINTS or cerebellar signs may represent a subgroup warranting high suspicion for central pathology and urgent neuroimaging. Adoption of such a selective approach may improve diagnostic accuracy and reduce inappropriate imaging overutilization.

## Data Availability

Data for this manuscript was obtained from network patients and is not publicly available because of patient privacy concerns.
